# A Study on the Clustering of Extra Virgin Olive Oils Extracted from Cultivars Growing in Four Ionian Islands (Greece) by Multivariate Analysis of Their Phenolic Profile, Antioxidant Activity and Genetic Markers

**DOI:** 10.3390/foods10123009

**Published:** 2021-12-04

**Authors:** Iliana Kalaboki, Dionysios Koulougliotis, Dimitra Kleisiari, Eleni Melliou, Prokopios Magiatis, Adamantia Kampioti, Effimia Eriotou, Aspasia Destouni

**Affiliations:** 1 Laboratory of Pharmacognosy and Natural Products Chemistry, Department of Pharmacy, National and Kapodistrian University of Athens, 15774 Athens, Greece; ilianakalaboki@yahoo.gr (I.K.); emelliou@pharm.uoa.gr (E.M.); magiatis@pharm.uoa.gr (P.M.); 2 Department of Environment, Ionian University, 29100 Zakynthos, Greece; dkoul@ionio.gr (D.K.); akampiot@ionio.gr (A.K.); 3 Department of Food Science and Technology, Ionian University, 28100 Argostoli, Kefalonia, Greece; dim.kleis1404@gmail.com (D.K.); eeriotou@ionio.gr (E.E.); 4 Laboratory of Cytogenetics and Molecular Genetics, Faculty of Medicine, University of Thessaly, 38221 Volos, Greece

**Keywords:** secoiridoid derivatives, antioxidant activity, genotyping, RAPD, Ionian Islands

## Abstract

Background: The phenolic fraction of extra virgin olive oil (EVOO) has disease preventive and health-promoting properties which are supported by numerous studies. As such, EVOO is defined as a functional food. The aim of the present study was to characterize the phenolic profile of olive oil from cultivars farmed in the Ionian Islands (Zakynthos, Kefalonia, Lefkada, and Kerkyra) and to investigate the association of phenols to antioxidant activity, which is central to its functionality. Furthermore, the study investigates whether multivariate analyses on the concentration of individual biophenolic compounds and genetic population diversity could classify the olive oil samples based on their geographic origin. Methods: Phenols were determined in 103 samples from different Ionian Island tree populations by ^1^H nuclear magnetic resonance (NMR), and sample antioxidant activity was measured by their capacity to reduce the free radical 2,2-diphenyl-1-picrylhydrazyl) (DPPH). Genetic diversity was measured by estimating Nei’s population genetic distance using 15 reproducible bands from random amplified polymorphic DNA (RAPD) genotyping. Results: Principal component analysis (PCA) of the secoiridoid concentrations clustered samples according to cultivar. Clustering based on genetic distances is not concordant with phenolic clustering. A cultivar effect was also demonstrated in the association between the concentration of individual phenols with DPPH reducing activity. Conclusions: Taken together, the study shows that the olive oil phenolic content defines “cultivar-specific phenolic profiles” and that environmental factors other than agronomic conditions contribute more to phenotype variance than genetics.

## 1. Introduction

Extra virgin olive oil (EVOO) is produced today mostly in Spain, Greece, and Italy (FAOSTAT data 2018) [1], https://www.fao.org/faostat/en/#data/QCL (accessed on 21 November 2021), Figure S1). Its health associated features have been attributed to its chemical composition, comprising over 200 determined chemical compounds. These include highly abundant (98–99% of the total weight) fatty acids such as monounsaturated acids (MUFA), e.g., oleic acid, as well as other low abundance bioactive compounds associated with beneficial effects to human health, such as phenolics, phytosterols, tocopherols, and squalene (1–2% of the total weight) [2,3]. Olive oil polyphenols have been implicated in the management of several chronic debilitating diseases. Studies in in vitro models have shown that polyphenolic compounds have anticancer and anti-inflammatory properties by regulating gene expression. Experimental results point to an association between the antioxidant properties and the exhibited biological activity of the polyphenolic compounds [4,5]. Furthermore, olive oil phenols have shown a protective effect on neurodegenerative disorders, with the most common ones being dementia, Alzheimer’s, and Parkinson’s diseases [6,7]. Virgin olive oil consumption reduces the risk of all-cause mortality, cardiovascular events, and stroke [8]. Due to the accumulating research on the beneficial and disease preventive activity of its phenolic compounds (approximately 2500 published reports to date [9]), EVOO is considered a functional food [10,11].

Based on credible scientific evidence supporting the health-promoting properties of EVOO, the European Food Safety Authority (EFSA) approved health claims for the “maintenance of normal blood cholesterol levels” when “replacing saturated fats in the diet with unsaturated fats”; “oleic acid is an unsaturated fat” [12,13]. As such, the European Union (EU 432/2012) distinguishes olive oils in terms of their effect on health depending on their phenolic content. An olive oil can be characterized as a “health-protecting food product” when its phenolic content exceeds 250 mg/kg. Specifically, the phenolic substances of olive oil that should be measured in order to support the health claim are mainly hydroxytyrosol and its secoiridoid derivatives (oleuropein and tyrosol complex); a variety of compounds related to oleuropein and ligstroside like oleacein, oleocanthal [14], oleuropein, and ligstroside; and aglycons [15] and their dialdehydic, monoaldehydic, and enolic forms (known also as oleuropeindials, ligstrodials, oleomissional, and oleokoronal) [16]. Recently, oleaceinic and oleocanthalic acid were added to the list of secoiridoid acids that are considered standard components of EVOO [17,18]. Therefore, the characterization and quantitative determination of olive oil polyphenols is extremely important to stakeholders [19], and as a result there has been an increased interest in the systematic characterization of the total concentrations of polyphenols, or of the individual compounds, with special attention given to the presence of oleacein and oleocanthal in olive oils mainly from Spain, Italy, and Greece [15,20,21,22,23,24,25].

Numerous factors affect the phenolic concentration of extra virgin olive oil, such as olive cultivar and fruit maturation, agronomic and environmental factors, processes used for olive oil extraction, packaging conditions, storage conditions, and shelf-life [20,26,27,28]. The above factors introduce variation to the phenolic profile of olive oils extracted even from the same cultivar. In addition, several analytical factors further complicate the comparison of the phenolic composition between olive oils. These include on one hand the numerous different methods used to determine and quantify the phenols, such as gas chromatography coupled with mass spectrometry; high performance liquid chromatography with electrochemical, mass spectrum (MS), ultraviolet (UV), diode array, or fluorescence detectors; ^31^P-NMR (nuclear magnetic resonance) and ^1^H-NMR spectroscopy [15,20,21,26,28,29,30]; and on the other hand, non-uniformity in the concentration units which are reported mainly as caffeic acid equivalents, gallic acid, or tyrosol equivalents. Therefore, the accurate determination of the secoiridoid compounds remains challenging.

Consequently, there has been an increase in the implementation of multivariate statistical methods on multiple biochemical and biophysical numerical data to identify which characteristics could serve as quality and composition identity “markers” between EVOOs from cultivars of different origin. In a previous study by our group, it was shown that discrimination of olive oil by geographical origin was possible by implementing multivariate analyses on the volatile compound profiles of olive oils extracted from the Ntopia cultivars from four different Ionian Islands [31]. The aim of the present study is first to characterize the differences of individual secoiridoid derivative levels between the olive oil samples from cultivars grown on four Ionian Islands and to determine cultivar-specific phenolic profiles. Further to the volatile profile study, the present work aims to investigate whether multivariate analyses on the concentration of individual biophenolic compounds could also discriminate between the olive oil samples based on their geographic origin. Finally, the study aims to investigate the genetic diversity between the different Ionian Island cultivars and their relationship to the phenolic profiles.

## 2. Materials and Methods

### 2.1. Olive Fruit Collection and Olive Oil Extraction Process

**Figure 1:**
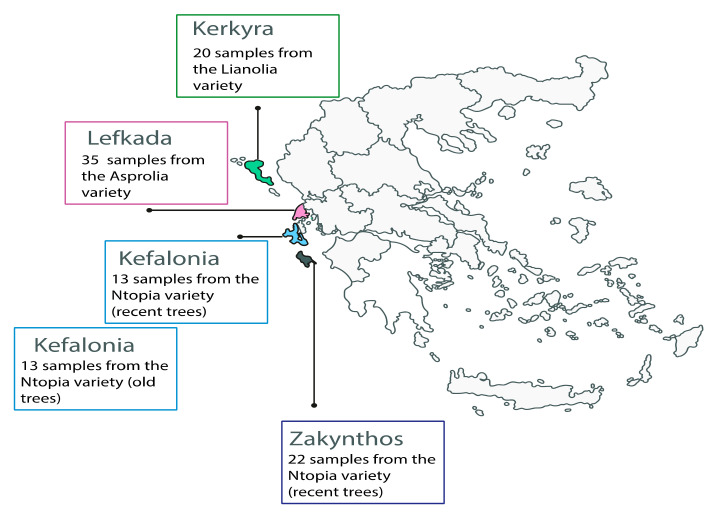
Map of Greece illustrating the geographical origin of the Ionian Island cultivars.

Inclusion in the study was based on the selection criteria described in our previous work [31]. Briefly, 103 extra virgin olive oil (EVOO) samples were extracted from hand-picked olive fruits sp. *Olea europaea* during the early harvest period (October) of the year 2017. Each of the 103 samples corresponds to one extraction from multiple immature, healthy, green, and firm fruits (monocultivar EVOO) from 35 Lefkada Asprolia, 22 Zakynthos Ntopia, 26 Kefalonia Ntopia (13 old and 13 recent trees), and 20 Kerkyra Lianolia trees (Figure 1, Table S1).

The selected study cultivars are planted on the Ionian Islands of Kerkyra (39°35′28.60″ N 19°51′50.54″ E; variety: Lianolia), Lefkada (38°43′ N 20°39′ E; variety: Asprolia), Kefalonia (38°15′54″ N 20°33′09″ E; variety: Ntopia), and Zakynthos (37°48′ N 20°45′ E; variety: Ntopia) (Table S1). Tree age was determined by measuring the perimeter of the trunk (including the bark) for the Ntopia variety of Kefalonia, at a height of 120 cm from the ground (Table S1). Kefalonia trees were classified as old and recent trees, and were analyzed as separate populations (Figure S2). The rationale for this *a priori* sub-categorization is based on research on Italian and Spanish cultivars, showing that cultivated ancient trees are genetically distinct from cultivated younger trees [32]. Region, climate conditions, and cultivar information have been previously described [31].

Fruit samples were stored in open containers at 4 °C and transferred within 24 h to the laboratory. Only mechanical methods were used to obtain olive oil in the laboratory. In brief, olives were crushed, pitted, ground, and malaxed for 30 min, as previously described [31]. Caution was taken so that the temperature during malaxation was always below 27 °C. Olive oil was extracted through centrifugation of each sample (4000 rpm for 5 min) and olive oil samples were stored in dark vials at −20 °C.

### 2.2. Sample Preparation for NMR Analysis

A quantity of 5.0 g of olive oil was mixed with 20 mL cyclohexane and 25 mL acetonitrile. The mixture was homogenized by a vortex mixer for 1 min and centrifuged for 5 min at 4000 rpm. 25 mL of the acetonitrile phase was collected and mixed with the internal standard consisting of 1.0 mL of a syringaldehyde solution (0.5 mg/mL) in acetonitrile, and evaporated under reduced pressure using a rotary evaporator (BUCHI, Flawil, Switzerland). The residue was dissolved in 750 μL CDCl_3_ and 550 μL of the solution was transferred to a 5 mm NMR tube.

### 2.3. NMR Spectral Analysis

^1^H NMR spectra were recorded at 400 MHz using a Bruker 400 NMR spectrometer. Typically, 50 scans were collected into 32 K data points over a spectral width of 0–16 ppm with a relaxation delay of 1 s and an acquisition time of 1.7 s. Prior to Fourier transformation (FT), an exponential weighting factor corresponding to a line broadening of 0.3 Hz was applied. The spectra were phased, corrected, and integrated automatically using TOPSPIN. When necessary, accurate integration was performed manually for the peaks of interest. Standard solutions and calibration curves were prepared as previously described in Dimantakos et al. [27]. Raw values are presented in Table S1.

### 2.4. Determination of Antioxidant Activity

The antioxidant activity of the olive oil samples was determined via measurement of their capacity to reduce the free radical DPPH (2,2-diphenyl-1-picrylhydrazyl), as adapted recently [33,34]. Thus, a quantity of 6 μL of the olive oil sample was added into 3 mL of a 100 μΜ freshly prepared stock solution of DPPH in ethyl acetate, and subsequently, the decay of the DPPH absorbance at 517 nm followed over a time period of 60 min. As explained previously [33,34], the total antioxidant activity of each olive oil sample was expressed in Trolox equivalents (TEAC in mM Trolox/L of olive oil) after fitting the DPPH absorbance time decay with a two-exponential equation, as shown below, by using five fitting parameters (I_F_, A_1_, A_2_, t_1_ and t_2_):

I(t) = I_f_ + A_1_ × exp(−t/t_1_) + A_2_ × exp(−t/t_2_) (1)

In Equation (1), I(t) is the DPPH absorbance at 517 nm at time t, I_f_ is the final DPPH absorbance value which is expected to be attained after an “infinite” amount of time which allows for expression of the total antioxidant activity of the olive oil sample, A_1_ and A_2_ are the amplitudes of each of the two exponentials, while t_1_ and t_2_ are their corresponding time constants. Experiments were repeated three times in a Shimadzu UV 2100 UV-VIS spectrophotometer at room temperature. DPPH and Trolox were obtained from Sigma-Aldrich, (Merck/MilliporeSigma, Burlington, MA, USA) and ethyl acetate was purchased from Merck (Merck/MilliporeSigma, Burlington, MA, USA).

Raw values are presented in Table S1.

### 2.5. DNA Extraction

DNA was extracted by ethanol precipitation, as previously described [35]. Briefly, dry leaves were cut into small pieces with a surgical blade, and were ground using a porcelain mortar and pestle in 5 mL of the extraction buffer (1% SDS, 0.5 M NaCl (Sigma-Aldrich, Merck/MilliporeSigma, Burlington, MA, USA)) for 10 min. The homogenates were transferred into 1.5 mL microfuge tubes and were stored at −20 °C overnight. Subsequently, the samples were subjected to a series of ultra-centrifugation (at 12,000 rpm for 10 min) and DNA purification steps, as described in the published method [35]. After air-drying the pellet for 3 h at room temperature to preclude ethanol precipitation, DNA was diluted in 50 μL ddH_2_O and sample concentration and purity were determined in a UV spectrophotometer by measuring absorption at 260 nm and calculating the 260/280 and 260/230 ratios. The samples were stored at −20 °C until further processing.

### 2.6. Random Amplified Polymorphic DNA (RAPDS)

To screen for polymorphic alleles across the *O. europaea* cultivar genome, 5 decamer oligonucleotide sequences were selected from the Eurofins Genomics 10mer RAPD kits v.02.10.2014 (Eurofins Genomics GmbH, Ebersberg bei München, Germany). Selection was based on their ability to amplify polymorphic alleles in *O. europaea*, as previously reported [36] (Supplementary Table S2). Polymerase chain reactions were performed with DreamTaq™ (Thermo Fisher Scientific, Inc., Waltham, MA, USA) DNA polymerase (5 U/μL) (Thermo Fisher Scientific, Inc., Waltham, MA, USA) in a final volume of 20 μL, containing 1× of 10× DreamTaq™ Buffer, 0.2 mM dNTPs, 0.25 U of polymerase, and 400 nM primer. Amplification was performed by denaturing DNA and activating the polymerase at 94 °C for 2 min, followed by 45 cycles comprising a dissociation step at 95 °C for 1 min, annealing at 34 °C for 1 min, and an extension step at 72 °C for 2 min followed by a final extension step at 72 °C for 5 min. Allele sizes were resolved by electrophoresis in a 2.5% agarose gel (1 × TAE) stained with SYBR™ Safe DNA Gel Stain (Sigma-Aldrich, Merck/MilliporeSigma, Burlington, MA, USA)in 100 V for 2 h. Image analysis was performed with the GelAnalyzer v19.1 software (http://www.gelanalyzer.com/?i=1 (accessed on 15 June 2019)).

### 2.7. Genetic Distance Estimation

The magnitude of genetic similarity between the cultivar varieties (populations) was estimated by calculating Nei’s genetic distance estimators [37]. Of the 103 samples, 61 with complete genotype and biochemical data corresponding to the 5 populations under investigation (Table S3) were analyzed. Briefly, allele frequencies were calculated as: p = number of present bands at a specific locus (allele-predetermined size) across all the samples in the population divided by the number of alleles, which in this case equals the number of samples. This is because the RAPDS method yields one band or its absence per locus and this is interpreted as haploidy. Then, q (alternative allele) frequency = 1 − p. Genetic distance was estimated as follows: Nei I (Nei genetic Identity) = Jxy/(JxJy)^0.5^ and Nei D (Nei genetic Distance) = −Ln(I), where Jx and Jy are the sum of the squared allele x and allele y frequencies, respectively, across populations, and Jxy is the sum of the product of the squared allele frequencies across populations. All calculations were performed by implementing the allele frequency calculation functions of the GenAlEx v6.51b2 excel add-in for haploid binarized genotypes [38]. The binarized genotype matrix (1 = presence of a band at the pre-determined allele size and 0 = absence of the corresponding band) was used as input. Nei’s Distances (D) were also clustered (“average linkage” = UPGMA) and visualized as a dendrogram with the hclust R package functions. The statistical certainty of each cluster was tested in pvclust R [39], where *p*-values are calculated through two types of tests: multiscale bootstrap resampling which calculates the AU (Approximately Unbiased) *p*-value and a *p*-value returned by normal bootstrapping. The *p*-value of a cluster is a value between 0 and 1, indicating the strength of cluster support by the data.

### 2.8. Multivariate Statistical Analyses

Differences in the composition of the phenolic compounds between cultivars were tested with a one-way analysis of variance (ANOVA) and Tukey’s post hoc test for multiple comparisons. Statistical significance was set at *p* < 0.05. A principal components analysis (PCA) was implemented to identify which variables, i.e., individual phenolic compounds, contribute the most to the variance between cultivars, i.e., which combinations of variables can discriminate the cultivar varieties, and also identify putative correlations between the variables (redundancy in discriminating between the cultivar varieties). PCA was run on scaled data (standard deviation = 1 and mean = 0) by invoking the *prcomp*() R v4.0.3 (2020-10-10) built-in function. Functions of the factoextra v1.0.6 and the ggplot2 v3.3.3 [40], R v4.0.3 (2020-10-10) packages were implemented to extract and plot the PCA results, such as the eigenvalues and the percent (%) contribution of each variable to each dimension (PC). Variables with significant contribution to the first and second principal components were determined as the ones with greater than the expected average contribution. Assuming that variable contributions were uniform, the expected average percent contribution would be 1/length (variables) = 1/7 = 14.3%. Therefore, for a given component, a variable with a contribution larger than this threshold could be considered as important in contributing to the component.

Pearson’s r was calculated to test the effect of the concentration of individual phenolic compounds on antioxidant activity per cultivar group. For all statistical tests the level of significance was set at *p* = 0.05 [41].

## 3. Results and Discussion

### 3.1. BioPhenolic Compound Concentrations of Olive Oils from Ionian Island Cultivars

**Figure 2:**
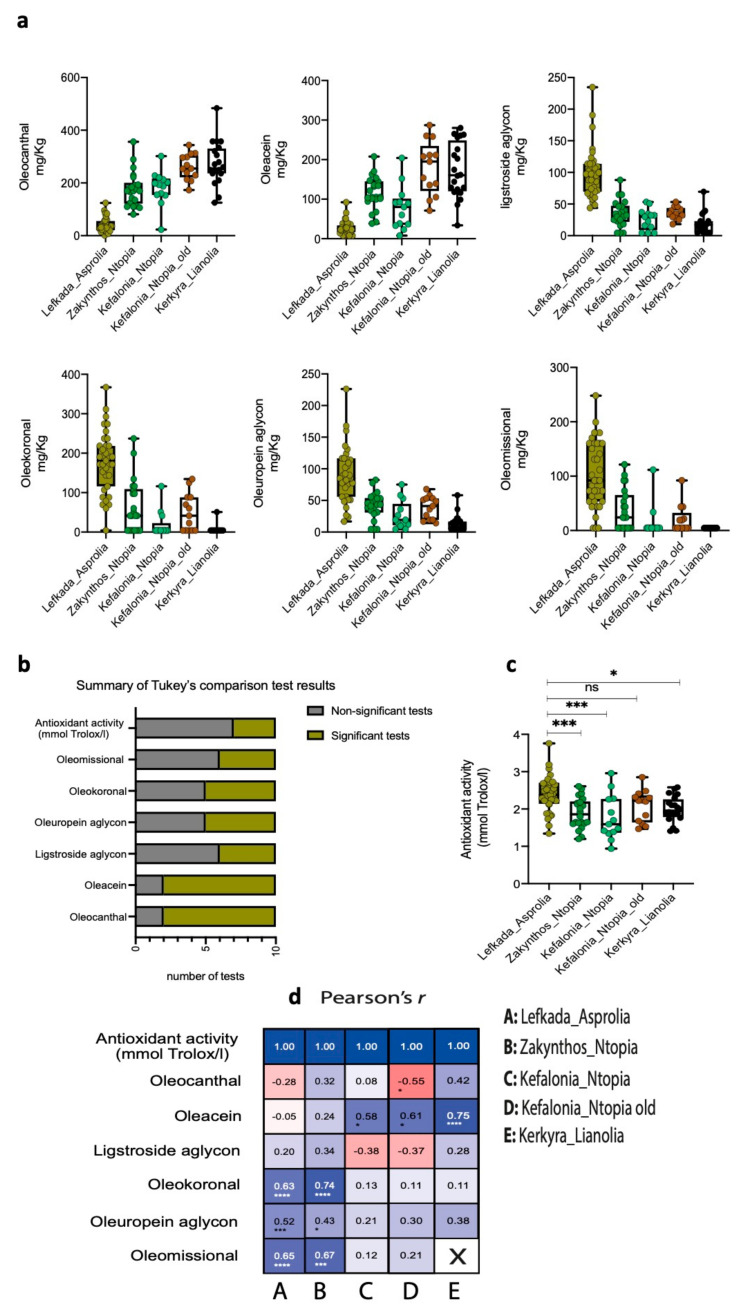
Cultivar effects on phenolic concentrations and antioxidant activity: (**a**) Box-plots illustrate phenolic compound concentration distributions. Each point represents a sample. Whiskers illustrate the minimum and maximum values; (**b**) bar-plots illustrate the number of significant and non-significant one-way ANOVA tests (Tukey’s pairwise comparisons); (**c**) box-plots of the distribution of the antioxidant activity values—each sample is represented by a single point. ns: non significant; (**d**) Pearson’s r coefficient matrix—blue colors illustrate positive correlations and red colors illustrate inverse (negative) correlation between phenolic compounds and antioxidant activity. *p*-values are illustrated with asterisks: * *p* ≤ 0.05, *** *p* < 0.01, **** *p* < 0.0001. X: values are identical and cannot be used in the linear model.

The analyzed olive oil samples were produced from monocultivar extractions, where each cultivar represents a distinct geographical region with the exception of Kefalonia _Ntopia, which is divided into two subgroups (populations) based on tree age (Figure 1). The formation of subgroups was motivated by evolutionary and ecological predictions, as well as morphological differences in the fruit. Descriptive statistics comprising the mean values, the standard deviation (SD), and the standard error of the mean (SEM) for each of the characterized phenolic substance concentrations and the antioxidant activity per cultivar are presented in Table 1. Concentrations for oleocanthal, oleacein, ligstroside aglycon, oleuropein aglycon, oleokoronal, and oleomissional were determined by NMR, as described in Section 2.2 and Section 2.3. The highest mean oleocanthal concentration was observed for the olive oil samples extracted from Kerkyra_Lianolia trees (273.5 mg/Kg) followed by Kefalonia_Ntopia_old trees (261.5 mg/Kg), and the lowest mean concentration was obtained for the samples from the Lefkada_Asprolia trees (Table 1, Figure 2a). The same trend was observed for oleacein. The Kerkyra_Lianiolia mean values are in line with those reported recently by Diamantakos et al. [20], who studied 5674 samples from over 30 Greek varieties.

The reverse trend was obtained for the rest of the secoiridoid derivatives under investigation where the Asprolia _Lefkada variety had the highest mean concentration values (Table 1, Figure 2a). Trend reciprocity, as well as differences in the phenolic profile between cultivars in our analysis, could indicate the effect of malaxation or the effect of the variety/location on the biophenolic concentrations in the extracted olive oil. Indeed, in an elegant recent study investigating the effect of tree variety, harvest time, malaxation duration, and temperature on the changes in secoiridoid derivative abundance, all factors were shown to influence biophenol abundance [27]. Interestingly, reciprocity in the concentration change of individual secoiridoid derivatives was observed in association to the duration of malaxation. Both oleocanthal and oleacein increased with malaxation time, whereas oleomissional and oleokoronal reached their peak concentration at the very early stages of malaxation and decreased thereafter. Based on the reciprocity of the concentration changes, the authors postulated that oleocanthal and oleacein are the products of the enzymatic transformation of oleokoronal and oleomissional, respectively [27]. The same study also showed that the changes in the individual biophenol’s abundance during malaxation differs between the four cultivars under identical extraction conditions. Similar to our observations for Lefkada_Asprolia, the cultivar Olympia showed that oleokoronal and oleomissional remained the major constituents of olive oil compared to oleocanthal and oleacein after 60 min of malaxation.

Based on the above, and since in the present study the harvest, ripening stage, and olive oil extraction conditions were maintained as identical for the different island cultivars, we attributed the observed phenolic compound concentration trends to a “variety/cultivar effect”. To further investigate the cultivar effect on the concentration of individual phenols, we performed one-way ANOVA with Tukey’s tests for pairwise comparisons. The significantly different tests per secoiridoid derivative and antioxidant activity are summarized in Figure 2b and Figure S3, in Table S3. In specific, Tukey’s multiple comparison test (total number of pairwise comparisons per biophenolic compound and antioxidant activity = 10) showed that oleocanthal and oleacein were the secoiridoid derivatives with the most significantly different pairwise tests between cultivars, with only two of the ten comparisons yielding non-significant results (Figure 2a,b and Figure S3, Table S2). Non-significant oleocanthal and oleacein tests were obtained for Kefalonia_Ntopia compared to Zakynthos_Ntopia, and Kerkyra_Lianolia compared to Kefalonia_Ntopia_old. Interestingly, Kefalonia_Ntopia_old samples and Kerkyra_Lianolia samples were not significantly different in any of the tests, probably indicating that these cultivars have similar phenolic biosynthetic processes despite their geographic distance.

We also observed that based on the pairwise tests two groups of phenolic substances could be formed. The first group is formed by oleomissional and ligstroside aglycon (number of significant tests = 4) and the second is formed by oleokoronal and oleuropein aglycon (number of significant tests = 5) (Figure 2b and Table S3). In specific, for both groups the significant differences were attributed to the comparison of Lefkada_Asprolia with all the other four cultivars. In addition to the Lefkada_Asprolia driven differences, oleokoronal and oleuropein aglycon differed significantly between Zakynthos_Ntopia and Kerkyra_Lianolia. These results probably reflect cultivar driven differences between the rate of enzymatic transformations of oleuropein or ligstroside to oleocanthal and oleacein through the oleuropein aglycon and ligstroside aglycon intermediates and the recently described oleokoronal and oleomissional.

Altogether, our observations are in line with the proposed cultivar differences described by Diamantakos et al. [27], where the authors showed that both the rate of the biosynthetic transformation leading to oleocanthal and oleacein and their yield differ between cultivars of different geographic origin.

### 3.2. Cultivar Effect on the Relationship between Olive Oil Antioxidant Activity and Individual Secoiridoids

Phenolic compounds act as antioxidants though radical scavenging, hydrogen atom transfer, and metal-chelation. Antioxidant activity has been shown to differ between olive oils produced from different cultivars [42]. Based on previous data and the observed Ionian Island differences between the individual phenol concentrations we reasoned that the cultivar driven phenolic olive oil profile differences would underlie differences in their capacity to reduce the free radical DPPH. A one way ANOVA with Tukey’s test for multiple testing showed that only the olive oil from Lefkada_Asprolia olives had significantly higher antiradical activity compared to Zakynthos_Ntopia, Kefalonia_Ntopia, and Kerkyra_Lianolia, but not from Kefalonia_Ntopia_old (Figure 2c and Figure S3). All other pairwise comparisons were not statistically significant (Figure S3). Since more statistical tests yielded significant results when we investigated the cultivar effect on individual phenolic compound concentrations, we reasoned that antioxidant activity would not be stably associated with the antiradical properties of a specific secoiridoid. The cultivar effects on the relationship between individual secoiridoids and the antioxidant activity measured in olive oils were reported by Montano et al. [43]. In specific, the group reported that none of the tested phenolics were stably highly correlated with all eight Spanish cultivars in separate correlation tests and that secoiridoid derivatives were highly correlated with only two of the total eight varieties. To investigate this effect in the Ionian Island samples we tested the correlation between secoiridoid levels and antioxidant activity per Ionian cultivar. In line with Monteno et al. [33], we also observed that none of the secoiridoids stably correlated with all cultivars (Figure 2d). Surprisingly, oleocanthal was not strongly and significantly correlated with the antioxidant activity of any of the studied olive oils. We also observed two trends; oleacein was quite highly correlated with the antioxidant activity of three olive oils, namely Kerkyra_Lianolia, Kefalonia_Ntopia, and Kefalonia_Ntopia_old. The compounds oleuropein aglycon, oleokoronal, and oleomissional display a moderate to relatively high correlation with the antioxidant activity of the other two olive oils, i.e., Lefkada_Asprolia and Zakynthos_Ntopia. Based on the findings reported by Montano et al. [43] (and those of other investigators cited therein), the association between EVOO biophenol compound concentration and antioxidant activity is characterized by increased complexity. Importantly, Montano et al. showed that the effect of the phenolic compounds on oxidative stability is low for the varieties with high oleic acid and low linoleic acid concentrations. In the same study, in three of the studied varieties with low oleic acid, antioxidant activity was influenced by the phenol, contributing to >50% of the total phenol concentration.

Taken together, our results indicate that differences in the phenolic content are more likely to allow cultivar classification than the antioxidant activity alone.

### 3.3. Multivariate Analysis of the Phenolic Profile Clusters Samples by Cultivar Geographic Origin

**Figure 3:**
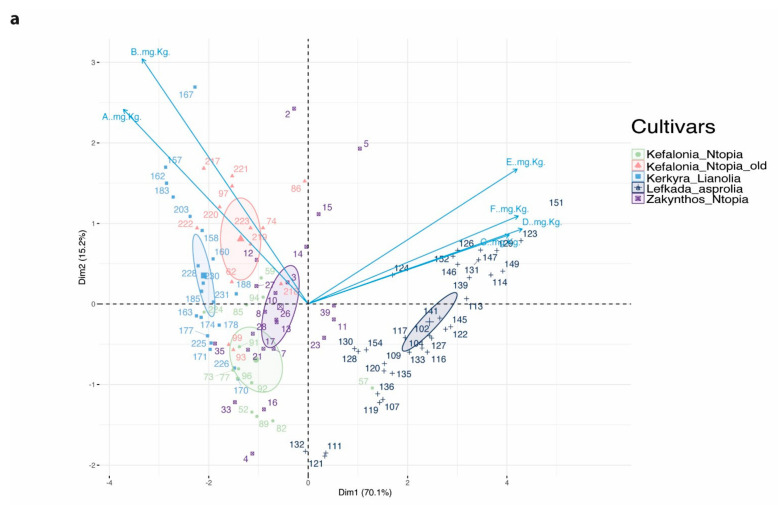

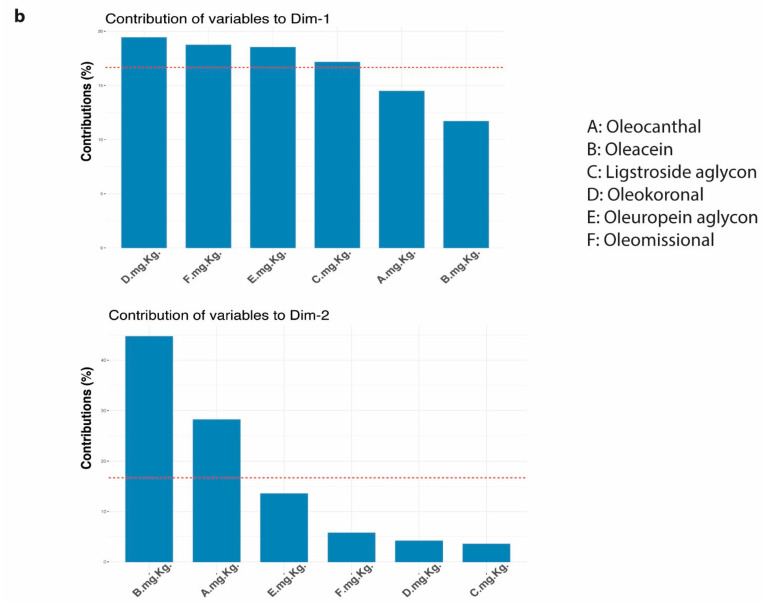
Principal component analysis for the phenolic substances of the olive oil for different Ionian Island cultivars. (**a**) Biplot of principal component for scores for olive oil samples of five cultivars to phenolic compounds. Confidence ellipses of 95% are drawn with the corresponding cultivar colors. The blue arrows illustrate variable eigenvectors. Dim-1: principal component 1 (PC1). Dim-2: principal component 2 (PC2). (**b**) Bar-plots show the proportion of each variable’s contribution to PC1 (**top**) and PC2 (**bottom**).

The pairwise comparisons reported in this study are indicative of cultivar-specific phenolic profiles for the samples produced from the four Ionian Islands. We next sought to investigate which combinations of individual phenolic compounds contribute the most to the observed differences between cultivars. To this end we implemented a principal component analysis and found that the cumulative cultivar variance explained by the first two principal components is 85.3%. Therefore, variation in the measured secoiridoid concentration explains most of the variation between the cultivars in the present study. The first principal component (PC1) explains 70.1% and is driven by the difference between Lefkada_Asprolia and Kerkyra_Lianolia, illustrated as the most distant clusters on the *x*-axis on the PCA biplot (Figure 3a). The first dimension also separates Lefkada_Asprolia from the other four cultivars, which are located on the opposite negative side of the *x*-axis. The 95% confidence ellipses plotted around the group means (Figure 3a) indicate that the olive oil samples under investigation can be clustered with confidence into distinct populations corresponding to the different cultivars. An overlap exists between the Kefalonia_Ntopia and the Zakynthos_Ntopia ellipses, reflecting the similarity of those olive oil samples regarding the secoiridoid concentrations driving both dimensions. The phenolic compounds driving the clustering, i.e., separating the samples in cultivar groups with high confidence, are the ones contributing the most to PC1 (Figure 3b). Interestingly, the compound with the highest contribution is oleokoronal, followed by oleomissional and the aglycons of oleuropein and ligstroside, but not oleocanthal and oleacein (Figure 3b). Therefore, the compounds that according to Diamantakos et al. [20,27] act as intermediates of the biochemical transformation pathway from oleuropein and ligstroside to oleocanthal and oleacein, are the most important secoiridoids for distinguishing between olive oil samples from the five specific Ionian Island cultivars. In contrast, this analysis suggests that the yield of their end products, namely oleocanthal and oleacein, is not a major contributor to PC1. Therefore, the present study suggests that the origin (cultivar/geographic origin) of the olive oil is mostly associated with the rate of the enzymatic transformations reflected in the concentrations of intermediates and not so with the concentration of oleocanthal or oleacein. Indeed, oleocanthal and oleacein contribute most to the second principal component (PC2), which explains only 15.2% of the variation between the Ionian Island cultivars.

The same analytical approach has been applied in Italian cultivars by Losito et al. [44], where the authors performed PCA on the secoiridoid measurements in cultivars from different locations under the same extraction conditions to investigate the cultivar/location effect on phenolic compound variation. They also observed cultivar clustering driven by differences in the secoiridoid concentrations. However, their samples came from geographical locations which are distant and probably have different climates. In the present study, all four islands are classified as “Csa” (hot dry-summer climates) according to the Köppen–Geiger climate classification system, as previously described in Eriotou et al. [31]. Therefore, the present study provides stronger evidence for the association between cultivar and secoiridoid concentration for the Ionian Island cultivars.

### 3.4. Ionian Island Cultivar Genetic Diversity Based on RAPD Genetic Markers

Since the multivariate analysis demonstrates that cultivars can be distinguished based on the concentration of intermediates in the biochemical transformation towards oleocanthal and oleacein, we next investigated the effect of genetics on cultivar phenotypic diversity. To do so, we analyzed a subset of the dataset comprising 61 samples with complete genetic and biochemical data.

**Figure 4:**
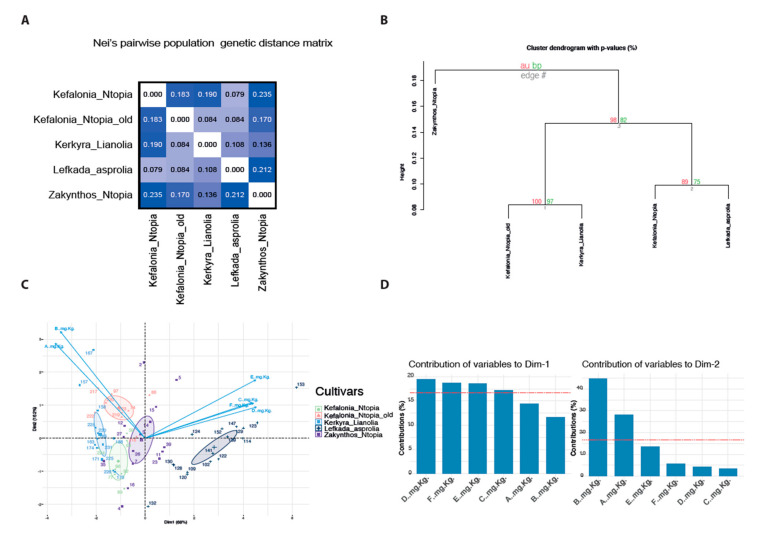
Genetic distance clustering between the Ionian Island cultivars: (**A**) Nei’s population genetic distance matrix; (**B**) dendrogram illustrating unsupervised hierarchical clustering of Nei’s genetic distance. Grey numbers: the order of cluster during agglomerative cluster construction. Red values on top of each branch: multiscale bootstrap resampling which calculates the AU (Approximately Unbiased) *p*-value. Red values on top of each branch: normal bootstrapping *p*-value. *p*-values are represented by percentages, e.g., 97: 97% certainty represents a *p*-value = 0.03; (**C**) PCA biplot of the phenolic compounds from sub-sampled 62 olive oils; (**D**) bar-plots representing the proportion of the contribution of each variable to PC1 and PC2.

PCR amplifications using a single RAPD primer pair produced 15 reproducible bands (Supplemental Data Table S3: Genotype Data), which were interpreted as haploid alleles. Polymorphism was defined as the percentage of alleles with frequencies other than one or zero, which represent the presence or absence of a band (allele) across all the samples of a population (cultivar), respectively. The populations with the highest percentage of polymorphic loci are Kerkyra_Lianolia and Lefkada_Asprolia (100%). Overall, the percentage of polymorphic loci was >80% (mean 93.33%, SE 2.98%, Table S3). Allele frequencies were used to calculate Nei’s population genetic distance estimate D. This metric represents the expected proportion of non-shared alleles between two populations. The highest pairwise genetic distances were observed for Kefalonia_Ntopia and Zakynthos_Ntopia (0.235), and for Zakynthos_Ntopia compared to Lefkada_Asprolia (0.212) (Figure 4A). The lowest distance was observed between Kefalonia_Ntopia_old and Kerkyra_Lianolia. Unsupervised hierarchical clustering with average linkage was implemented to cluster the cultivars based on their genetic similarities. The first cluster connects Kefalonia_Ntopia and Lefkada_Asprolia with high certainty based on the *p* values (Figure 4B). The second cluster is formed by Kerkyra_Lianolia and Kefalonia_Ntopia_old, also with high certainty. The only variety that forms a single branch is Zakynthos_Ntopia (Figure 4B). PCA was performed on the phenolic profiles from the subset of the 61 samples to investigate whether this subset recapitulates the sample clustering observed for the 103 samples (Figure 3c). The analysis on the smaller sample subset produced similar results with the PCA on the 103 samples (Figure 3a,b and Figure 4C,D), supporting extrapolation to the larger dataset.

When considering hierarchical clustering for the genetic distances and the PCA for the phenolic compounds, we observed that the sample clustering is not concordant. For example, cultivar differences driven by phenolic profiles are most profound for Kerkyra_Lianolia compared to Lefkada_Asprolia. However, these cultivars have only a moderate pairwise genetic distance compared to the distance between Lefkada_Asprolia and Zakynthos_Ntopia. The only concordant comparison involves the similarity observed between Kefalonia_Ntopia_old and Kerkyra_Lianolia. The concentrations of biophenolic compounds and the genetic distances clustered the samples from these cultivars very closely on the PCA biplots, and the ANOVA tests were non-significantly different.

Similar results to ours were reported in a recent study by Omri et al. [45], who observed a lack of similarity between the clusters generated from genetic data, compared to physicochemical data. In specific, they calculated Nei’s gene diversity h from 36 RAPD and 94 ISSR polymorphic bands, and clustered 19 Tunisian cultivars. They observed that the most accurate dendrogram was obtained when information from both types of markers was combined, as opposed to the dendrograms constructed using either marker group. Importantly, they also observed that when they clustered the cultivars by combining information from both physicochemical and genetic marker data, the resulting dendrogram clusters were more similar to the ones obtained using only RAPD and ISSR data than to the physicochemical driven groupings in their PCA analysis. They concluded that the genetic markers are more robust in cultivar classification compared to physicochemical markers.

To the best of our knowledge, this is the first study reporting Ionian Island cultivar classification based on genetic markers. Our results indicate that Kerkyra_Lianolia and Kefalonia_Ntopia_old trees are more genetically identical, and this similarity expands to the similarity (non-statistically significant differences) in the phenolic compound concentrations in EVOOs from those trees. In addition, our results also indicate that the concentration of olive oil phenolics is a phenotype that is greatly influenced by the environment, decreasing the proportion of variation explained by genetics (heritability). Moreover, quantitative traits are complex and therefore controlled by interactions between multiple genes and the environment. In the livestock breeding sector, field and herd management is significantly optimized to reduce environmental variation and improve the estimation of heritability. The advent of innovative high-throughput genotyping technologies such as genotyping-by-sequencing has greatly improved the identification of genetic markers that associate with specific traits [46]. These technologies yield high density genotypes by profiling highly informative (single nucleotide polymorphisms) SNPs, which serve as quantitative trait loci (QTL) associated with economically important traits. Based on the progress evidenced in *Agrigenomics*, in other crop and livestock sectors we anticipate that molecular marker assisted phenolic profiling will be a robust and cost-effective approach to identify and classify EVOOs with health claims. Cataloguing informative single nucleotide variants in olive trees is still in its infancy [47,48,49] and our work highlights the need to intensify the research and development of high-throughput olive genotyping platforms.

## 4. Conclusions

The present study represents the first investigation of the phenolic content in olive oils produced from Ionian Island cultivars under tightly controlled agronomic conditions. Multivariate analyses showed that the olive oil phenolic content defines “cultivar-specific phenolic profiles” and can be further investigated as a classifier by expanding the repertoire of measured secoiridoids. Interestingly, antioxidant activity was not stably correlated with a specific phenolic compound across cultivars, likely reflecting the effect of the cultivar on the complexity of the biochemical mechanisms mediating free radical DPPH reduction. This finding has implications for the development of methods to identify EVOOs with high antioxidant activity so that its functionality can be stably preserved. Finally, in accordance with previous reports, RAPD genotype-based cultivar classification did not reflect phenolic profile sample clustering. This highlights the need to develop novel and high-throughput genotyping platforms and to optimize agronomic practices to reduce environmental variation. This will allow quantitative trait association mapping and, ultimately, marker assisted EVOO classification.

## Figures and Tables

**Table 1:** Descriptive statistics for the analytical parameters per cultivar.

	Lefkada_Asprolia (n = 35)			Zakynthos_Ntopia (n = 22)			Kefalonia_Ntopia (n = 13)			Kefalonia_Ntopia_old (n = 13)			Kefrkyra_Lianolia (n = 20)		
	Mean	SD	SEM	Mean	SD	SEM	Mean	SD	SEM	Mean	SD	SEM	Mean	SD	SEM
Antioxidant activity (mmol Trolox/L)	2.393	0.4781	0.08081	1.893	0.4119	0.08782	1.773	0.5961	0.1653	2.086	0.4234	0.1174	1.994	0.3436	0.07684
Oleocanthal (mg/Kg)	39.47	27.26	4.607	176.2	65.48	13.96	181.7	64.74	17.96	261.5	49.22	13.65	273.5	81.47	18.22
Oleacein (mg/Kg)	27.04	17.6	2.975	115.2	41.6	8.869	78.42	55.15	15.3	179.5	68.19	18.91	172	70.35	15.73
Ligstroside aglycon (mg/Kg)	97.98	40.17	6.79	35.08	21.06	4.49	25.78	16.62	4.61	35.27	9.708	2.692	19.19	15.99	3.575
Oleuropein aglycon (mg/Kg)	178	78.82	13.32	64.38	66.73	14.23	26.49	22.58	6.263	38.85	18.02	4.997	6.627	10.41	2.327
Oleokoronal (mg/Kg)	90.56	42.91	7.254	41.65	21.13	4.506	19.34	33.03	9.16	50.12	47.83	13.27	14.92	13.03	2.913
Oleomissional (mg/Kg)	102.5	61.33	10.37	37.77	37.62	8.021	15.06	30.14	8.36	20.87	25.8	7.155	4.57	0	0

## Data Availability

Raw data (phenol concentrations and antioxidant activity measurements) are presented in Supplementary Table S1 which accompanies the manuscript.

## Supplementary Materials

The following are available online at https://www.mdpi.com/article/10.3390/foods10123009/s1, Figure S1: virgin olive oil production in the European Union (27)-FAOSTAT Data 2018, Figure S2: student’s *t*-test for the trunk perimeter of old and recent Kefalonia trees, Figure S3: summarized results of the pairwise Tukey’s tests, Table S1: raw measurements (phenols, Trolox, and tree perimeter), Table S2: RAPD primers, Table S3: genetic analysis.
