# Supplementary material for: A Study on the Clustering of Extra Virgin Olive Oils Extracted from Cultivars Growing in Four Ionian Islands (Greece) by Multivariate Analysis of Their Phenolic Profile, Antioxidant Activity and Genetic Markers
Source: Foods. 2021 Dec 4;10(12):3009. doi: 10.3390/foods10123009 (PMC8700953; doi:10.3390/foods10123009)
Supplement: Supplementary file 1 [file foods-10-03009-s001.zip › Figure S2.pdf]

Supplementary Figure S2

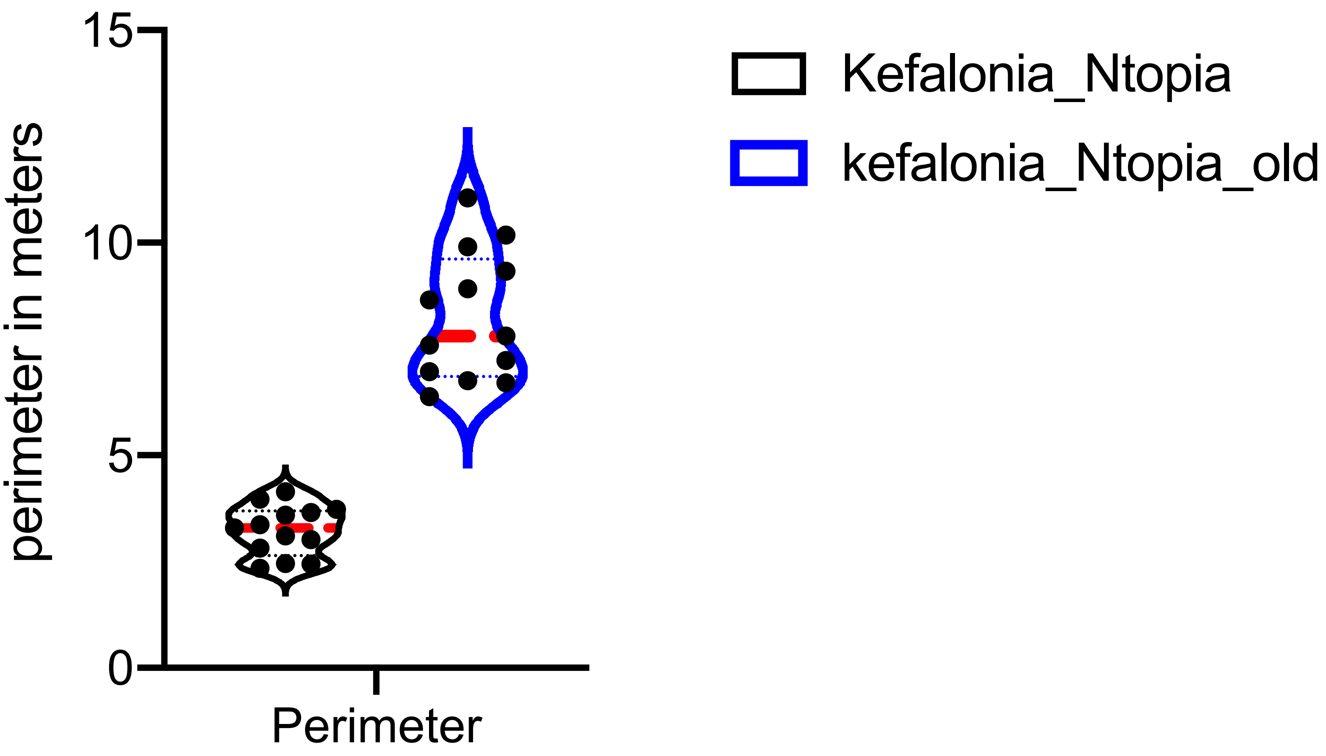

Paired t test	
P value	<0.0001
P value summary	****
Significantly different (P < 0.05)?	Yes
One- or two-tailed P value?	Two-tailed
t, df	t=15.18, df=12
Number of pairs	13
How big is the difference?	
Mean of differences (B - A)	5.042
SD of differences	1.197
SEM of differences	0.3321
95% confidence interval	4.318 to 5.765
R squared (partial eta squared)	0.9505
How effective was the pairing?	
Correlation coefficient (r)	0.6749
P value (one tailed)	0.0057
P value summary	**
Was the pairing significantly effective?	Yes
