# Supplementary figures and images for: A Study on the Clustering of Extra Virgin Olive Oils Extracted from Cultivars Growing in Four Ionian Islands (Greece) by Multivariate Analysis of Their Phenolic Profile, Antioxidant Activity and Genetic Markers
Source: Foods. 2021 Dec 4;10(12):3009. doi: 10.3390/foods10123009 (PMC8700953; doi:10.3390/foods10123009)

Supplementary Figure S1

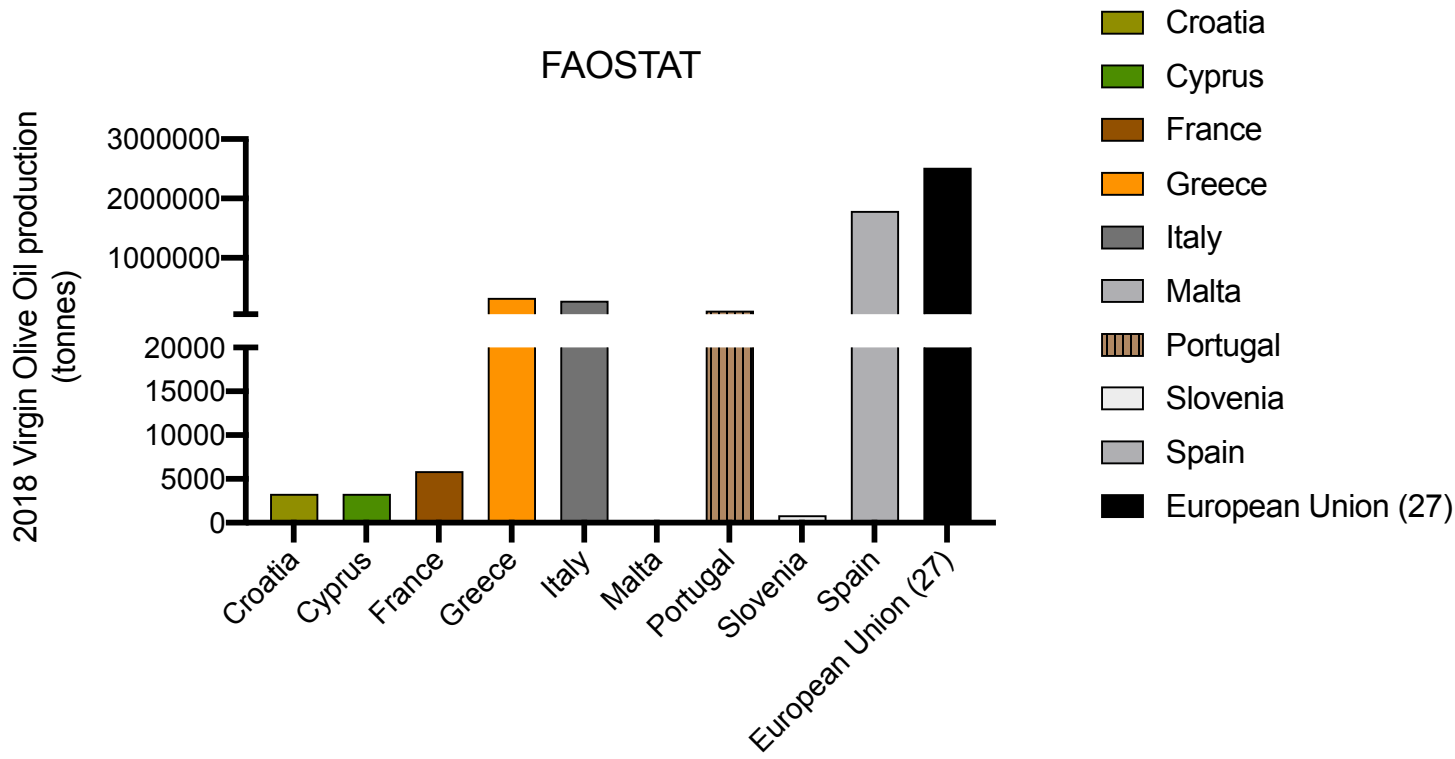

Supplement: Supplementary file 1 [file foods-10-03009-s001.zip › Figure S1.pdf]

Supplementary Figure S3

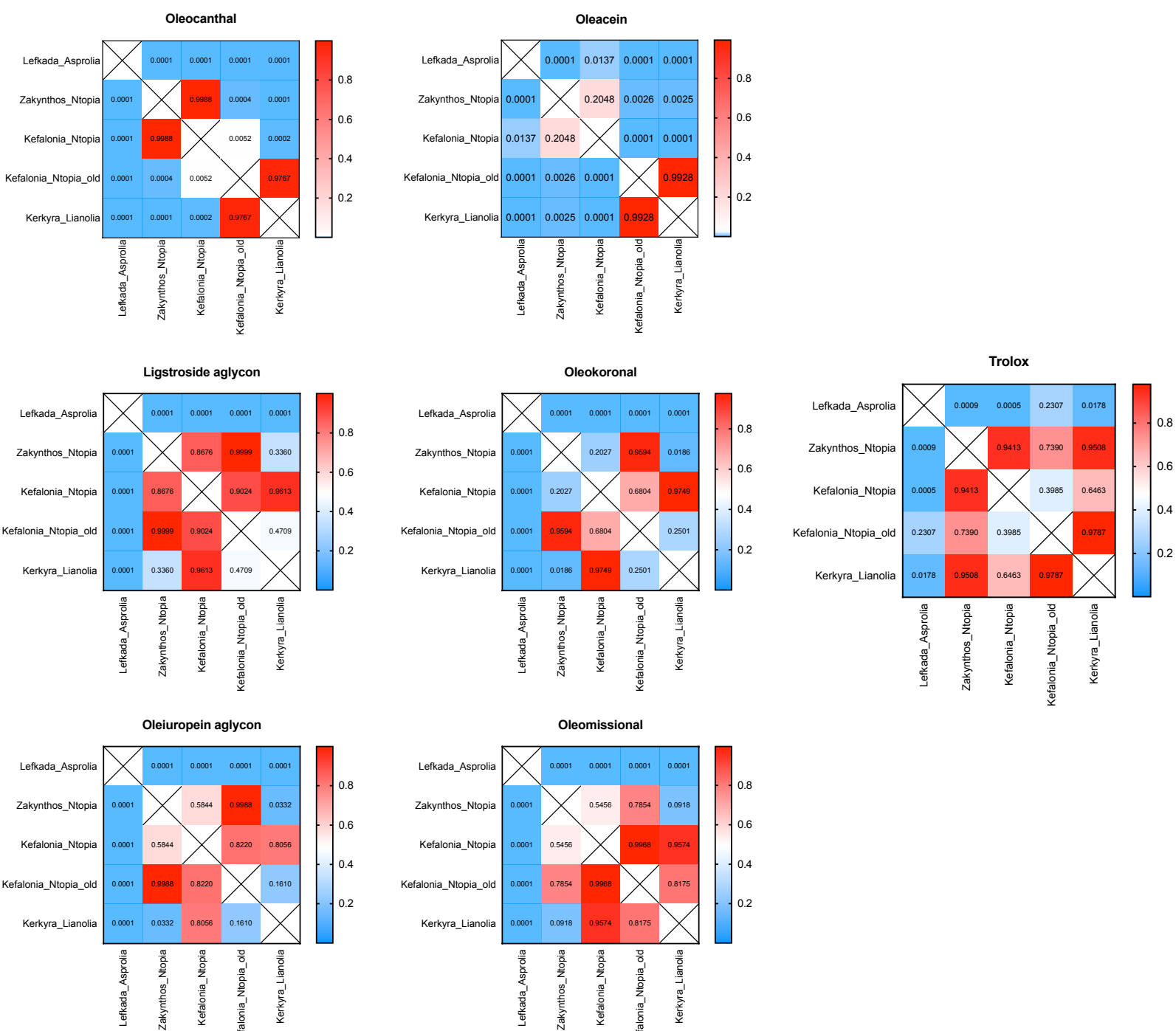

Supplement: Supplementary file 1 [file foods-10-03009-s001.zip › Figure S3.pdf]
